# Arterial hypertension in infants with congenital diaphragmatic hernia following surgical repair

**DOI:** 10.1007/s00431-024-05509-3

**Published:** 2024-04-06

**Authors:** Clara Engel, Judith Leyens, Bartolomeo Bo, Lennart Hale, Hannah Lagos Kalhoff, Lotte Lemloh, Andreas Mueller, Florian Kipfmueller

**Affiliations:** 1 Department of Neonatology and Pediatric Intensive Care, Children’s Hospital, University of Bonn, Venusberg-Campus 1, 53127 Bonn, Germany; 2 Center for Rare Diseases Bonn, Division of Congenital Malformations, University Hospital Bonn, Bonn, Germany

**Keywords:** Congenital diaphragmatic hernia, Extracorporeal membrane oxygenation, Arterial hypertension, Blood pressure, Follow-up

## Abstract

Pulmonary hypertension (PH) and cardiac dysfunction are established comorbidities of congenital diaphragmatic hernia (CDH). However, there is very little data focusing on arterial hypertension in CDH. This study aims to investigate the incidence of arterial hypertension in neonates with CDH at hospital discharge. Archived clinical data of 167 CDH infants who received surgical repair of the diaphragmatic defect and survived for > 60 days were retrospectively analyzed. Blood pressure (BP) values were averaged for the last 7 days before discharge and compared to standard BP values for sex, age, and height provided by the AHA in 2004. BP values reaching or extending the 95th percentile were defined as arterial hypertension. The use of antihypertensive medication was analyzed at discharge and during hospitalization. Arterial hypertension at discharge was observed in 19 of 167 infants (11.3%) of which 12 (63%) were not receiving antihypertensive medication. Eighty patients (47.9%) received antihypertensive medication at any point during hospitalization and 28.9% of 152 survivors (*n* = 44) received antihypertensive medication at discharge, although in 45.5% (*n* = 20) of patients receiving antihypertensive medication, the indication for antihypertensive medication was myocardial hypertrophy or frequency control. BP was significantly higher in ECMO compared to non-ECMO patients, despite a similar incidence of arterial hypertension in both groups (13.8% vs. 10.1%, *p* = 0.473). Non-isolated CDH, formula feeding, and minimal creatinine in the first week of life were significantly associated with arterial hypertension on univariate analysis. Following multivariate analysis, only minimal creatinine remained independently associated with arterial hypertension.

*Conclusion*: This study demonstrates a moderately high incidence of arterial hypertension in CDH infants at discharge and an independent association of creatinine values with arterial hypertension. Physicians should be aware of this risk and include regular BP measurements and test of renal function in CDH care and follow-up.

**Table:** 

**What is Known:** *• Due to decreasing mortality, morbidity is increasing in surviving CDH patients.* *• Pulmonary hypertension and cardiac dysfunction are well-known cardiovascular comorbidities of CDH.*	
**What is New:** *• There is a moderately high incidence of arterial hypertension in CDH infants at discharge even in a population with frequent treatment with antihypertensive medication.* *• A more complicated hospital course (ECMO, higher degree of PH, larger defect size) was associated with a higher risk for arterial hypertension.*

**What is Known:**

*• Due to decreasing mortality, morbidity is increasing in surviving CDH patients.*

*• Pulmonary hypertension and cardiac dysfunction are well-known cardiovascular comorbidities of CDH.*

**What is New:**

*• There is a moderately high incidence of arterial hypertension in CDH infants at discharge even in a population with frequent treatment with antihypertensive medication.*

*• A more complicated hospital course (ECMO, higher degree of PH, larger defect size) was associated with a higher risk for arterial hypertension.*

## Introduction

Congenital diaphragmatic hernia (CDH) is a complex developmental malformation of the newborn. It is defined as a defect in the diaphragm, enabling abdominal structures to herniate into the thoracic cavity [1]. This leads to prenatal compression of the fetal lung, pulmonary hypoplasia, and cardiac, musculoskeletal, and neurodevelopmental implications [2]. CDH affects about 2.4 to 4.1 newborns per 10,000 births [1]. In recent years, survival rates have continuously increased, and mortality is currently estimated to be 20–40% [3–5]. However, improved survival rates are associated with a higher morbidity [1, 2, 5]. Several studies have discussed developmental and therapeutic approaches of pulmonary hypertension (PH) in neonates with CDH [6–8]. In contrast, there are only very little data on arterial hypertension in the context of CDH.

Arterial hypertension in CDH is supposed to derive from different developmental and iatrogenic causes. Pulmonary vascular changes, proliferation of smooth muscle cells, and a reduced number of alveoli in CDH might contribute to the development of arterial hypertension, as has been similarly shown for preterm infants with bronchopulmonary dysplasia (BPD) [9–11]. In both, CDH and BPD, these changes are associated with the development of PH and arterial hypertension [2, 12].

CDH is the most common indication for extracorporeal membrane oxygenation (ECMO) in neonatal respiratory failure [13] and arterial hypertension has been associated with the use of ECMO [12, 14]. Vasopressors, inotropes, steroids, and other medications frequently utilized in infants with CDH are known to cause transient arterial hypertension as an adverse effect [15–18]. Besides, arterial hypertension is one of the short-term consequences of acute kidney injury (AKI), which has been shown to occur more frequently in CDH neonates [19, 20].

The importance of evaluating and treating arterial hypertension in CDH infants is demonstrated by the numerous long-term consequences of arterial hypertension, including reduced cardiovascular function in childhood and adolescence, facilitating cerebrovascular and renal disease, hypertensive retinopathy, and left ventricular hypertrophy [21, 22]. Additional studies have shown a correlation between arterial hypertension during childhood and the development of cardiovascular risk factors in adulthood [23].

This study aims to investigate the incidence of arterial hypertension in neonates with CDH at the time of discharge from hospitalization.

## Methods

### Study design and variables

We performed a retrospective analysis of archived clinical data of CDH infants treated at our institution over a 10-year period (2012–2021). Exclusion criteria were as follows: mortality within 60 days of life, late-presenting CDH (diagnosis > 24 h of life), major cardiac defects (need for cardiac surgery or catheter intervention within 60 days), and non-retrievable patient charts. Infants with minor cardiac defects (atrial septal defect, persistent foramen ovale, ventricular septal defect) were included. We reviewed demographic and clinical data, including defect size and location, observed-to-expected lung-to-head-ratio (o/e LHR), time of operation, incidence, and severity of PH and cardiac dysfunction, proBNP values (pg/ml), use and duration of ECMO, prenatal diagnosis, duration of intubation and oxygen support, fetoscopic endoluminal tracheal occlusion (FETO), and feeding at discharge. PH severity was graded as < 2/3 systemic pressure (mild PH), 2/3 systemic pressure to systemic pressure (moderate PH), and suprasystemic pressure (severe PH). Cardiac function was assessed in accordance with current American Society of Echocardiography Guidelines utilizing qualitative and quantitative measures in a stepwise approach. Function was classified as normal, right ventricular (RV) dysfunction (indicated by, in order, global or regional cardiac hypokinesia, S’-wave < 5.0 cm/s on Tissue Doppler Imaging, tricuspid annular plane systolic excursion (TAPSE) < 0.7 cm, RV fractional area change ≤ 25%), left ventricular (LV) dysfunction (indicated by global or regional cardiac hypokinesia, fractional shortening ≤ 25%, ejection fraction ≤ 45%, or LV output < 100 ml/kg/min), or biventricular dysfunction (combination of LV and RV dysfunction) [24]. Acute kidney injury (AKI) was assessed using the RIFLE-criteria proposed by the Acute Dialysis Quality Initiative workgroup in 2004 [25]. RIFLE-criteria define AKI stages by the increase of creatinine compared to the lowest creatinine measured in the first week of life, greater then 48 h after birth (baseline creatinine). Stages are as follows: “Risk” (stage 1, increase of ≥ 25% from baseline creatinine), “Injury” (stage 2, increase of ≥ 50% from baseline creatinine), and “Failure” (stage 3, increase of ≥ 75% from baseline creatinine) [25]. We used the highest creatinine value measured during hospitalization for RIFLE-stage calculation, and creatinine values were measured in mg/dl. Additionally, the minimal creatinine value in the first week of life (day 3–day 7) and the maximum creatinine during the hospital stay were recorded and compared between patients with and without arterial hypertension.

### Blood pressure measurement and definition of arterial hypertension

Blood pressure (BP) was analyzed the last 7 days before hospital discharge. In patients receiving antihypertensive medication, BP was analyzed in 3 days before the respective medication was started. Mean values for systolic BP (BP_sys_), diastolic BP (BP_dia_), and mean BP (BP_mean_) of the last 7 days and the last day before discharge were calculated. All BP values referred to in this article are presented as mean BP values and have been calculated from at least six measurements. Additionally, the duration of treatment with the first-line BP medication was noted.

Arterial hypertension was defined according to the 2016 European Society of Hypertension guidelines using American Heart Association (AHA) data as average BP_sys_ reaching or extending the 95th percentile for sex, age, and height [26–28]. BP values between the 90th and 95th percentile were classified as high-normal [26–29]. BP was measured either oscillometric, with weight-appropriate-sized cuffs, or invasively via an arterial line. In infants with continuous invasive BP measurement, values were noted every 4 h. In infants with oscillometric BP measurement, up to six values per day were used for analysis. In case more than 3 of 7 days were measured oscillometric, the measurement was defined as oscillometric. Calculated mean BP values were compared to standard BP values for sex, age, and height provided by the AHA in 2004 [30], and categorized according to the respective percentiles. Categories, used for our study, were BP values < 90 percentile, ≥ 90 percentile, ≥ 95 percentile, and ≥ 99 percentile.

### Antihypertensive treatment strategies

Arterial hypertension was treated with diuretics, ß-blockers, calcium channel blockers, or angiotensin-converting-enzyme (ACE) inhibitors. Underlying and concomitant disorders were taken into consideration when deciding on the best fitting agent. Usually, infants with concomitant structural or functional cardiac disorders, such as myocardial hypertrophy and tachycardia, were treated with ß-Blockers to limit cardiac stress. In case of presumed volume overload, diuretics were the preferred treatment option. Infants without cardiac disorders were usually treated with calcium channel blockers, or ACE-inhibitors. All patients were carefully monitored for adverse effects.

### CDH-treatment protocol

Postnatal management was performed according to the CDH Euro Consortium treatment guidelines [31]. In general, a gentle lung-protective ventilation strategy was applied, with maximum peak inspiratory pressure (PIP) of 24–26 mmHg, positive end-expiratory pressure (PEEP) of 2–4 mmHg, and a respiratory rate of 60–80 per minute. Initially, inspired oxygen fraction (FiO_2_) was 1.0 and titrated to achieve a postductal partial oxygen pressure (PaO_2_) of 80–150 mmHg. Ventilator settings were adjusted to achieve a target pCO_2_ of 45–60 mmHg.

Infants were treated with inhaled nitric oxide (iNO) therapy to treat PH. If appropriate, infants received dobutamine or milrinone to treat cardiac dysfunction and norepinephrine and vasopressin to achieve MAP > gestational age in weeks. Fentanyl and midazolam were used for sedation as needed.

The indication for ECMO was evaluated according to the CDH Euro Consortium criteria [31]. For ECMO, the Deltastream DP3 system (Xenios, Aachen, Germany) was used [32].

Surgical repair was performed after clinical stabilization when pulmonary vascular resistance had decreased. In ECMO patients, surgical repair was usually performed after weaning from ECMO.

### Statistics

All analyses were performed using the IBM SPSS statistics software Version 27 (IBM Corp., Armonk, NY). Continuous variables were described using median and interquartile range (IQR), and categorical variables were summarized as absolute number (*n*) and percentage. Mann-Whitney *U* test was used to compare continuous variables and Pearson’s $\chi^{2}$ test and Fisher’s exact test for categorical covariates between patients with and without arterial hypertension.

Covariates that have been associated with worse outcome in patients with CDH in previous studies were tested in the univariate analysis [33–36]. To identify factors associated with arterial hypertension, univariate analysis was performed. After exclusion of colinear variables, only factors that were significantly associated with arterial hypertension on univariate analysis were included in multivariate logistic regression. Multicollinearity was tested using the variance inflation factor and Pearson correlation. Goodness of fit was assessed using the Hosmer-Lemeshow test. The area under the curve (AUC) was calculated using receiver operating characteristics (ROC) analyses of minimal and maximum creatinine for predicting arterial hypertension at discharge. Using ROC analysis, an optimal cutoff value of 0.5 mg/dl for minimal creatinine and of 0.79 mg/dl for maximum creatinine to predict arterial hypertension was identified. A *p*-value < 0.05 was considered significant.

## Results

### Cohort characteristics

Overall, 241 CDH neonates were treated in our institution between May 2012 and December 2021. Of these, 74 neonates were excluded for the following reasons: mortality within the first 60 days of life (*n* = 53), missing patient charts (*n* = 10), late-presenting CDH (*n* = 5), and congenital heart defect (*n* = 6), leaving a final study cohort of 167 infants.

Ninety-nine (59.3%) patients were male, mean gestational age (GA) was 37.5 weeks, and 15 infants (9.0%) deceased in-hospital > 60 days of life. A total of 162 patients (97.0%) underwent oscillometric BP measurements. In 7 days before discharge, 19 infants (11.3%) had BP_sys_ ≥ 95 percentile and 49 (29.3%) had BP_mean_ ≥ 95 percentile before discharge. Of the 15 patients, who deceased in hospital, 1 patient (6.7%) had BP_sys_ > 95 percentile in the last 7 days before they died. Overall, 28.9% of 152 survivors (*n* = 44) received antihypertensive medication at the time of discharge, and 47.9% of all included patients (80/167 patients) received antihypertensive medication for at least 1 week at any time during hospitalization. Of the 44 patients receiving antihypertensive medication at discharge, in 45.5% (*n* = 20), the indication for antihypertensive medication was myocardial hypertrophy or frequency control.

Antihypertensive medications used to treat arterial hypertension in our cohort included ß-blockers (*n* = 57), ACE-inhibitors (*n* = 9), diuretics (*n* = 46), calcium-channel-blockers (*n* = 9), and ⍺1-adrenoreceptor-antagonists (*n* = 4), including therapy with multiple agents.

### Arterial hypertension vs. no-hypertension patients

Infants with arterial hypertension (7-day BP_sys_) were compared according to their baseline and outcome characteristics with infants without systolic arterial hypertension (Tables 1, 2, and 3). There was no significant difference in patient demographics except for a higher proportion of non-isolated CDH in the arterial hypertension group (*p* = 0.034). There was no significant difference in ECMO rate and duration, or echocardiographic evidence of PH or cardiac dysfunction. Infants with arterial hypertension had an older age at repair (*p* = 0.005) and lower rates of breast milk feeding (*p* = 0.034).

**Table 1:** Comparison of demographics of patients with 7-day systolic arterial hypertension (BP_sys_ ≥ 95 percentile) and without 7-day systolic arterial hypertension (BP_sys_ < 95 percentile)

**Variables**		**No Hypertension (*n* = 148; 88.6%)**	**Hypertension (*n* = 19; 11.4%)**	***p*-value**
**Demographics**				
Gender, (male)		84 (56.8%)	15 (79.9%)	0.064
Gestational age, (weeks)		37.5 [37.1–37.9]	37.6 [36.8–38.5]	0.803
Birthweight, (kg)		3.0 [2.9–3.1]	3.1 [2.8–3.4]	0.929
Inborn		130 (87.8%)	17 (89.5%)	0.836
Prenatally diagnosed CDH		130 (87.8%)	18 (94.7%)	0.373
Left-sided CDH		130 (87.8%)	15 (78.9%)	0.281
Liver-up CDH		72 (48.6%)	12 (63.2%)	0.234
LHR, (%)		42.6 [40.3–45.0]	43.0 [35.6–50.3]	0.181
Isolated CDH		138 (93.2%)	15 (78.9%)	**0.034**
FETO		17 (11.5%)	4 (21.1%)	0.236
Defect size	A	14 (9.5%)	2 (10.5%)	0.261
	B	54 (36.5%)	3 (15.8%)	
	C	54 (36.5%)	8 (42.1%)	
	D	26 (17.6%)	6 (31.6%)	
Minor cardiac malformations	ASD/ PFO	35 (23.6%)	7 (36.8%)	0.212
	VSD	4 (2.7%)	1 (5.3%)	0.538

Confidence level 95%; values printed bold represent *p* < 0.05

*ASD* atrial septal defect, *CDH* congenital diaphragmatic hernia, *FETO* fetoscopic endoluminal tracheal occlusion, *LHR* lung-to-head-ratio, *PFO* persistent foramen ovale, *VSD* ventricular septal defect

*Only survivors

**Table 2:** Comparison of outcome parameters of patients with 7-day systolic arterial hypertension (BP_sys_ ≥ 95 percentile) and without 7-day systolic arterial hypertension (BP_sys_ < 95 percentile)

**Variables**		**No hypertension (*n* = 148; 88.6%)**	**Hypertension (*n* = 19; 11.4%)**	***p*-value**
**Outcome**				
PH medication at discharge^a^		85 (63.4%)	14 (77.8%)	0.230
Antihypertensive medication at discharge^a^		37 (27.6%)	7 (38.9%)	0.322
Antihypertensive medication at any point		77 (52.0%)	12 (63.2%)	0.360
ECMO		50 (33.8%)	8 (42.1%)	0.473
Repeat ECMO		5 (3.4%)	0 (0.0%)	0.416
Duration of ECMO		2.9 [1.8–4.1]	4.5 [0.5–8.6]	0.155
Age at ECMO-Start, (hours)		10.1 [5.3–15.0]	6.4 [1.8–11.0]	0.246
Age at repair, (days)		5.3 [4.8–5.8]	8.5 [4.4–12.5]	**0.005**
Mechanical ventilation, (days)		15.2 [11.6–18.8]	19.7 [12.5–27.0]	0.434
Oxygen support, (days)		29.2 [23.6–34.9]	63.7 [24.7–102.9]	0.503
CLD (oxygen support > 28 days), (percent)		57 (38.5%)	9 (47.4%)	0.457
Oxygen support at discharge		32 (21.6%)	5 (26.3%)	0.643
AKI	0	5 (26.3%)	13 (8.8%)	0.133
	1	3 (15.8%)	23 (15.5%)	
	2	4 (21.1%)	36 (24.3%)	
	3	7 (36.8%)	76 (51.4%)	
Days in hospital		54.1 [47.4–60.8]	94.1 [59.1–129.2]	0.529
In hospital mortality > 60 days		14 (9.5%)	1 (5.3%)	0.547
Feeding	Breast milk	92 (62.2%)	7 (36.8%)	**0.034**
	Formula feeding	51 (34.5%)	9 (47.4%)	0.270

Confidence level 95%; values printed bold represent *p* < 0.05

*CDH* congenital diaphragmatic hernia, *PH* pulmonary hypertension, *CLD* chronic lung disease, *ECMO* extracorporeal membrane

^a^Only survivors

**Table 3:** Comparison of hemodynamics of patients with 7-day systolic arterial hypertension (BP_sys_ ≥ 95 percentile) and without 7-day systolic arterial hypertension (BP_sys_ < 95 percentile)

**Variables**	**No hypertension (*n* = 148; 88.6%)**	**Hypertension (*n* = 19; 11.4%)**	***p*-value**
**PH**			
No PH	15 (10.1%)	3 (15.8%)	0.309
Mild PH	44 (29.7%)	2 (10.5%)	
Moderate PH	57 (38.5%)	10 (52.6%)	
Severe PH	32 (21.6%)	4 (21.1%)	
**Cardiac dysfunction**			
No cardiac dysfunction	58 (39.2%)	5 (26.3%)	0.276
Right ventricular cardiac dysfunction	45 (30.4%)	8 (42.1%)	0.302
Left ventricular cardiac dysfunction	4 (2.7%)	0 (0.0%)	0.468
Biventricular cardiac dysfunction	41 (27.7%)	6 (31.6%)	0.724
**Cardiac biomarkers**			
proBNP (pg/ml)	10,807 [4368–17,246]	11,477 [9048–13,905]	0.435

Confidence level 95%

*PH* pulmonary hypertension

When comparing infants with 7-day BP_mean_ ≥ 95 percentile and < 95 percentile, those with 7-day BP_mean_ ≥ 95 percentile more often received ECMO (*p* = 0.004) with longer runs (*p* = 0.050); had an older age at repair (*p* = 0.024), a higher degree of PH (*p* = 0.015), and incidence of chronic lung disease (CLD; defined as oxygen support > 28 days; *p* = 0.021); and were more frequently treated with antihypertensive medication at any point (*p* = 0.019).

Age at surgical repair was significantly higher for infants with a 7-day BP_sys_, as well as 7-day BP_mean_ ≥ 95 percentile (*p* = 0.005, *p* = 0.024) (Table 2).

### ECMO vs. non-ECMO patients

**Fig. 1:**
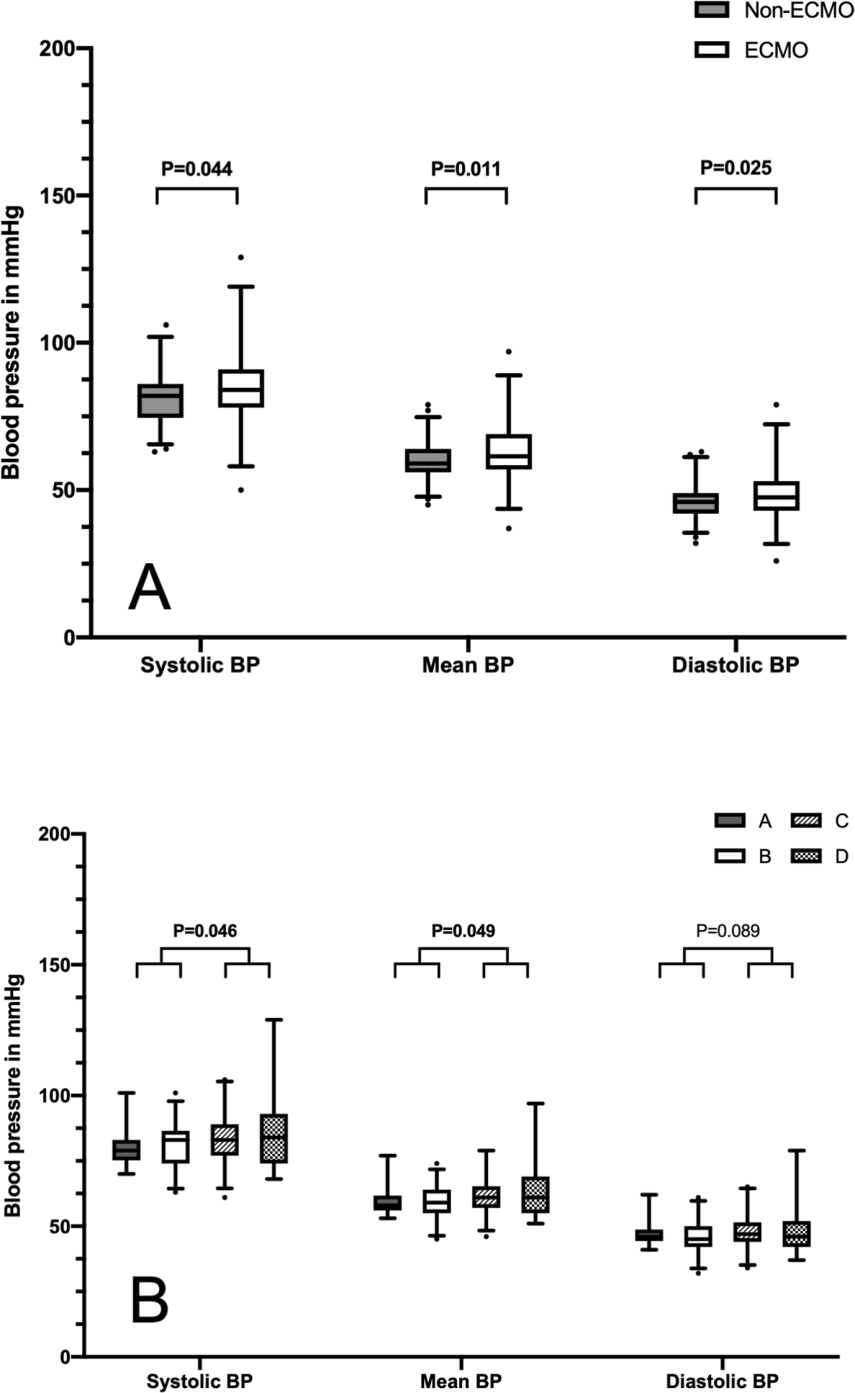
Box-plot comparison of systolic, mean, and diastolic BP in ECMO and non-ECMO-patients (**A**) and according to defect sizes (**B**). Values printed bold represent *p* < 0.05. Whiskers represent the 95% confidence interval. BP, blood pressure; ECMO, extracorporeal membrane oxygenation

Infants with and without ECMO were compared according to their baseline demographics, incidence of PH and cardiac dysfunction, and outcome parameters (Table 4). Infants that received ECMO more often had a 7-day BP_sys_ ≥ 90 percentile (*p* = 0.029) and a 7-day BP_mean_ ≥ 95 percentile. Overall BP_sys_, BP_mean_, and BP_dia_ were significantly higher in ECMO patients (Fig. 1a).

**Table 4:** Comparison of arterial hypertension and outcome parameters in ECMO vs. non-ECMO patients

Variables		Non-ECMO patients (*n* = 109; 65.3%)	ECMO patients (*n* = 58; 34.7%)	*p*-value
**Arterial hypertension (7-day mean)**				
BP_sys_ ≥ 90 percentile		15 (13.8%)	16 (27.6%)	**0.029**
BP_sys_ ≥ 95 percentile		11 (10.1%)	8 (13.8%)	0.473
BP_sys_ ≥ 99 percentile		5 (4.6%)	4 (6.9%)	0.529
BP_mean_ ≥ 90 percentile		50 (45.9%)	35 (60.3%)	0.075
BP_mean_ ≥ 95 percentile		24 (22.0%)	25 (43.1%)	**0.004**
BP_mean_ ≥ 99 percentile		7 (6.4%)	9 (15.5%)	0.057
**Outcome**				
PH medication at discharge^a^		57 (53.3%)	42 (93.3%)	** < 0.001**
Antihypertensive medication at discharge^a^		16 (15.0%)	28 (62.2%)	** < 0.001**
Antihypertensive medication at any point		42 (38.5%)	47 (81.0%)	** < 0.001**
Age at repair, (days)		4.1 [3.8–4.5]	8.6 [7.1–10.0]	** < 0.001**
Mechanical ventilation, (days)		9.0 [7.9–10.2]	29.9 [20.9–38.9]	0.430
Oxygen-support, (days)		19.0 [14.9–23.2]	67.2 [49.9–84.4]	0.394
CLD (oxygen support > 28 days), (percent)		21 (19.3%)	45 (77.6%)	** < 0.001**
Oxygen-support at discharge		10 (9.2%)	27 (46.6%)	** < 0.001**
Days in hospital		40.0 [34.2–45.7]	93.8 [79.8–107.8]	0.420
In hospital mortality > 60 days		2 (1.8%)	13 (22.4%)	** < 0.001**
Feeding^b^	Breast milk	77 (70.6%)	22 (37.95%)	** < 0.001**
	Formula feeding	36 (33.0%)	24 (41.4%)	0.284

Confidence level 95%; values printed bold represent *p* < 0.05

*BP* blood pressure, *CLD* chronic lung disease, *ECMO* extracorporeal membrane oxygenation, *BP*_*mean*_ mean arterial blood pressure, *PH* pulmonary hypertension, *BP*_*sys*_ systolic arterial blood pressure

^a^Only survivors

^b^Only survivors, feeding with formula and breast milk included, other feedings excluded

The use of antihypertensive medication during hospitalization and at discharge, as well as the use of cardiac medication, was more frequent in ECMO patients than in non-ECMO patients (*p* < 0.001, *p* < 0.001, *p* = 0.021). ECMO patients generally showed more severe CDH characteristics. Defect size, grade of PH, and incidence of cardiac dysfunction were significantly higher in ECMO patients (*p* < 0.001 each). ECMO patients more often underwent prenatal FETO (*p* < 0.001) and more often had a liver herniation (*p* = 0.021).

### Defect size and BP

We compared defect sizes A to D according to the Congenital Diaphragmatic Hernia Study Group (CDHSG) scale for grating defect sizes in CDH to BP_sys_, BP_mean_, and BP_dia_ [37] (Fig. 1b). Patients with a higher defect size (C/D) had significantly higher BP_sys_ (*p* = 0.046) and BP_mean_ (*p* = 0.049) compared to patients with a lower defect size (A/B).

### AKI and creatinine values

**Fig. 2:**
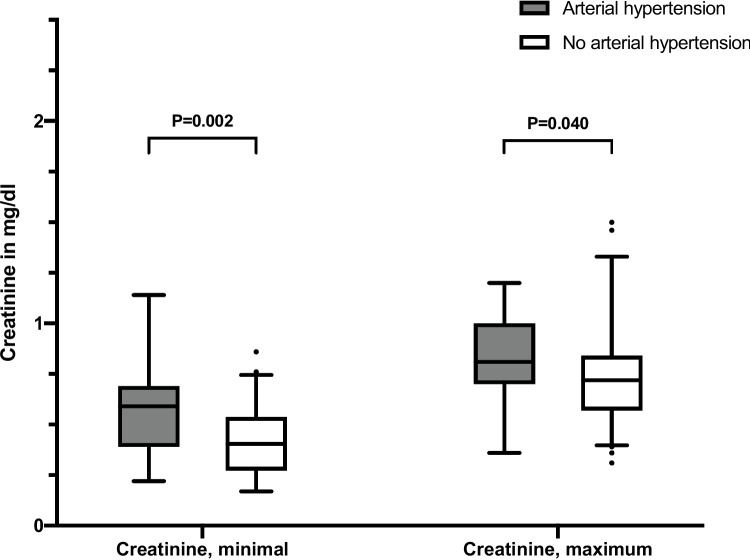
Box-plot comparison of minimal and maximum creatinine according to arterial hypertension. Values printed bold represent *p* < 0.05. Whiskers represent the 95% confidence interval

We reviewed AKI using the RIFLE-criteria proposed by the Acute Dialysis Quality Initiative workgroup, evaluating creatinine values throughout hospital stay [25]. According to these criteria, 18 (10.8%) of patients had no AKI, 26 (15.6%) patients had stage 1, 40 (24.0%) patients had stage 2, and 83 (49.7%) patients had stage 3 AKI at any point during hospitalization. Incidence and severity of AKI were not statistically associated to the incidence of arterial hypertension at hospital discharge (*p* = 0.133). However, minimal and maximum creatinine values were significantly higher in infants with compared to infants without arterial hypertension (Fig. 2). The AUC of minimal and maximum creatinine for predicting arterial hypertension was 0.716 (95% CI, 0.587–0.846; *p* = 0.001) and 0.645 (95% CI, 0.506–0.783; *p* = 0.041), respectively. Sensitivity and specificity of minimal creatinine > 0.5 mg/dl for predicting arterial hypertension were 63.2% and 71.6%, respectively. Positive and negative predictive values of minimal creatinine > 0.5 mg/dl were 22.2% and 93.8%, respectively. Sensitivity and specificity of maximum creatinine > 0.79 mg/dl for predicting arterial hypertension were 52.6% and 69.6%, respectively, with a positive predictive value of 18.2% and a negative predictive value of 92.0%.

### Univariate and multivariate analysis

On univariate analysis, non-isolated CDH, formula feeding, and minimal creatinine were significantly associated with the presence of arterial hypertension at discharge. Following multivariate analysis, minimal creatinine was each independently associated with hypertension (*p* = 0.002), while non-isolated CDH and formula feeding were not independently associated with hypertension (Table 5). Goodness-of-fit was assessed using the Hosmer-Lemeshow test, indicating a good model fit, $\chi^{2}$(8) = 6.14, *p* = 0.631.

**Table 5:** Uni- and multivariate analysis

**Variable**	**Univariate Analysis**		**Multivariate Analysis**	
	**Odds ratio (95% CI)**	**P-value**	**Odds ratio (95% CI)**	**P-value**
Gender, male	2.86 (0.91-9.02)	0.074		
Prenatal diagnosis	0.40 (0.05-3.19)	0.388		
Gestational age, days, mean (SD)	1.01 (0.97-1.04)	0.791		
Birth weight, g, mean (SD)	1.00 (1.00-1.000	0.395		
Non-isolated CDH	**3.68 (1.03-13.18)**	**0.045**	3.03 (0.77-11.86)	0.122
o/e LHR, %, mean (SD)	1.002 (0.971-1.04)	0.915		
Liver-up	1.81 (0.68-4.85)	0.239		
High-risk defect (C/D)	2.38 (0.82-6.95)	0.113		
Biventricular dysfunction	1.21 (0.43-3.38)	0.724		
PH >2/3 Systemic RR	1.86 (0.64-5.43)	0.258		
ECMO	1.43 (0.54-3.77)	0.475		
Chronic lung disease	1.28 (0.49-3.35)	0.610		
Formula feeding	**2.82 (1.05-7.57)**	**0.040**	2.55 (0.89-7.35)	0.082
Creatinine, minimal (mg/dl)	**8.55 (1.19-61.34)**	**0.033**	**8.9 (1.41-56.27)**	**0.020**
Creatinine, maximum (mg/dl)	2.52 (0.57-11.08)	0.221		

## Discussion

The incidence of arterial hypertension (defined as BP_sys_ ≥ 95 percentile) in the neonatal period ranges between 0.2 and 3% [18, 38–43]. In our cohort consisting of 167 CDH neonates, we observed an incidence of arterial hypertension of 11.3% (BP_sys_ ≥ 95 percentile), being noticeably higher than in the general neonatal population. To our knowledge, there are no comparable data in the CDH population.

We observed a significantly higher overall BP in patients with a history of ECMO support (Fig. 1b). This association has been described in multiple studies [12, 14]. Mechanisms which promote high BP in ECMO patients are volume overload, renal injury, and use of corticosteroids [14, 44]. Volume overload has a direct influence on BP and has often been reported in patients during their ECMO runs; however, our study demonstrates persistent arterial hypertension in ECMO patients at discharge [45–47]. The use of corticosteroids has been shown to be associated with arterial hypertension, which may result from increased systemic vascular resistance and intravascular volume, as well as inotropic effects of corticosteroids [14]. Systemic stress response to ECMO, subsequently leading to a catecholamine surge resulting in systemic peripheral vasoconstriction and in return a hyperdynamic cardiac function, is another possible cause of arterial hypertension in ECMO patients [14].

ECMO has shown to be associated with a higher prevalence of AKI in CDH neonates [48, 49], while AKI is associated with systemic hypertension in CDH [12, 20]. Zwiers et al. [49] observed that 64% of patients showed evidence of AKI during ECMO. Various factors are considered to facilitate AKI in ECMO, including altered renal perfusion resulting in reduced renal function and AKI [14]. In addition, CDH itself is considered to be a risk factor for AKI in neonates. Ryan et al. [20] showed an association of ECMO and AKI in CDH infants, with an incidence of AKI of 37%. In a recent study by Liberio et al. [48], AKI in neonates with CDH was associated with ECMO, use of diuretics, and abdominal closure surgery. Increased abdominal pressure after surgical CDH repair may harm kidney function due to altered renal perfusion, aggravating the risk of AKI [48]. In our study cohort, incidence and severity of AKI, defined by the RIFLE criteria, were not statistically associated with the incidence of arterial hypertension. However, minimal and maximum creatinine values were significantly higher in infants with arterial hypertension, although in uni- and multivariate analysis, only minimal creatinine in the first week of life was independently associated with the development of arterial hypertension, supporting a possible relation of renal injury and reduced renal function with the development of arterial hypertension in infants with CDH. This finding highlights the need for more future studies, investigating the association of renal function and short- and long-term outcome in CDH.

In our cohort, defect size was additionally associated with elevated BP_sys_ and BP_mean_ (Fig. 1b), which might be explained by a generally higher morbidity of infants with larger defect sizes. Putnam et al. demonstrated that defect size was the greatest predictor for overall morbidity, length of hospital stay, and duration of mechanical ventilation [50]. In our cohort, defect sizes were significantly higher in ECMO patients (*p* < 0.001). Ryan et al. [20] showed an association between defect size and the development of AKI. Both factors possibly influence the development of arterial hypertension.

We detected significantly lower BP_sys_ levels in infants receiving breastmilk compared to infants fed with formula (*p* = 0.034). El-Khuffash et al. [51] recently showed a significant increase in biventricular performance and an improvement in pulmonary artery acceleration time in preterm infants fed with breast milk, thereby indicating a possible protective effect of breastmilk on cardiac function and pulmonary hypertension. Although, breast milk has been linked to better lung development, improved cardiovascular health, and lower BP [52–54], it remains uncertain whether the protective effects of breast milk feeding suggested by our findings might have been biased by better CDH severity characteristics in infants receiving breast milk. This might result from a higher adherence to breast feeding in a shorter and less severe course of disease. This is supported by the significantly higher number of infants receiving breast milk in the non-ECMO group compared to the ECMO group (*p* < 0.001).

In our cohort, 44 (28.9%) patients were treated with antihypertensive medication at discharge. Of these, 7 (15.9%) patients demonstrated persistent arterial hypertension despite treatment, suggesting insufficient therapy. This underlines the importance of improving antihypertensive screening and treatment in CDH-patients.

Although arterial hypertension seems to be a significant comorbidity in surgically repaired CDH infants, studies investigating this issue are scarce [7, 8, 55, 56]. Neonatal arterial hypertension might be a transient problem in a significant subset of affected children; it may however persist well beyond infancy [38]. The importance of gaining knowledge about systemic effects of CDH cannot be underrated, due to the numerous long-term complications of arterial hypertension. In order to prevent these complications, regular BP measurements should be implemented in CDH follow-up programs.

Antihypertensive medication should be adjusted to achieve BP values of at least below the 95th, preferably the 90th percentile. In children, it is [26–29] recommended to start antihypertensive therapy with a low dose monotherapy of either ACE-inhibitors, ß-blockers, angiotensin II receptor blockers, calcium channel blockers, or diuretics [26–28]. ß-blockers are currently not regarded first line therapy but can be considered when appropriate [26, 27]. Even though not considered first line therapy, we made positive experiences with ß-blockers and favor them due to their cardioprotective effects [57].

In children at risk of heart failure and early cardiovascular events, such as children with end-stage renal failure, diabetes, or congenital heart disease, ACE inhibitors are considered first line therapy. If BP targets cannot be reached, they can be combined with low-dose beta-blockers. In patients with volume overload, the additional use of diuretics is advised [26, 28]. In CDH infants, cardiopulmonary health is often altered, and impairment often continues into adolescence and adulthood. In our cohort, 62.3% of patients had cardiac dysfunction, either uni- or biventricular. Kraemer et al. [58] assessed cardiopulmonary health in a group of adult CDH survivors and observed airflow obstruction, reduced lung volumes, and reduced exercise capacity, in ECMO patients compared to non-ECMO patients. These findings underline the importance of assessing cardiac function when treating hypertension in CDH patients.

Our study has some limitations that need to be mentioned. As a retrospective analysis, BP measurements were taken per standard nursing guidelines and not under controlled study conditions. BP values were averaged over 7 days, and only the average value was taken into consideration. Further, a substantial proportion of patients received antihypertensive medication at the time of hospital discharge, which might have led to an underestimation of the incidence of arterial hypertension in our population. Multivariate analysis was only performed for BP_sys_ values, while BP_mean_ values were also elevated in our study. We used a cut-off of ≥ 95th percentile, while it might be appropriate to consider lower cut-off values as well. Furthermore, we performed a single center retrospective study, only analyzing archived clinical data, resulting in the risk of center specific treatment-associated bias. The long study period of 10 years leaves the risk of historical bias due to possibly updated treatment options for both CDH and arterial hypertension.

To conclude, our study demonstrates that there is a moderately high incidence of arterial hypertension infants with CDH at discharge. In our cohort, a more complicated hospital course (ECMO, higher degree of PH, larger defect size) was established as a risk factor for arterial hypertension. Physicians need to be aware of regular BP measurements in hospital to adjust treatment of arterial hypertension accordingly. Further studies are required to evaluate the long-term course and consequences of arterial hypertension in CDH follow-up.

## Data Availability

Research data can be provided upon request by the corresponding author.

## Abbreviations

ACE: Angiotensin converting enzyme
AHA: American Heart Association
AKI: Acute kidney injury
BPD: Bronchopulmonary dysplasia
CDH: Congenital diaphragmatic hernia
CDHSG: Congenital Diaphragmatic Hernia Study Group
CLD: Chronic lung disease
ECMO: Extracorporeal membrane oxygenation
FETO: Fetoscopic endoluminal tracheal occlusion
GA: Gestational age
iNO: Inhalative nitric oxide
LV: Left ventricle
o/e LHR: Observed to expected lung to head ratio
PaO_2_: Postductal partial oxygen pressure
PH: Pulmonary hypertension
RV: Right ventricle
TAPSE: Tricuspid annular plane systolic excursion
